# tRNA‐derived fragments in gastric cancer: Biomarkers and functions

**DOI:** 10.1111/jcmm.17511

**Published:** 2022-08-11

**Authors:** Maryam Kohansal, Ali Ghanbarisad, Reza Tabrizi, Abdolreza Daraei, Mojtaba Kashfi, Hailin Tang, Cailu Song, Yongming Chen

**Affiliations:** ^1^ Department of Medical Biotechnology Fasa University of Medical Sciences Fasa Iran; ^2^ Department of biology Payame Noor University Tehran Iran; ^3^ Noncommunicable Diseases Research Center Fasa University of Medical Sciences Fasa Iran; ^4^ Department of Medical Genetics, School of Medicine Babol University of Medical Sciences Babol Iran; ^5^ Departmen of Microbiology, School of Medicine Shahid Beheshti Univercity of Medical Sciences Tehran Iran; ^6^ State Key Laboratory of Oncology in South China, Collaborative Innovation Center of Cancer Medicine Sun Yat‐sen University Cancer Center Guangzhou China; ^7^ Department of Gastric Surgery Sun Yat‐Sen University Cancer Center Guangzhou China

**Keywords:** biomarker, Gastric cancer, tRNA‐derived Fragments

## Abstract

tRNA‐derived fragments (tRFs), non‐coding RNAs that regulate protein expression after transcription, have recently been identified as potential biomarkers. We identified differentially expressed tRFs in gastric cancer (GC) and the biological properties of tRFs in predicting the malignancy status of GCs as possible biomarkers. Until 15 February 2022, two independent reviewers did a thorough search in electronic databases of Scopus, EMBASE and PubMed. The QUADAS scale was used for quality assessment of the included studies. Ten articles investigating the clinical significance of tRFs, including 928 patients, were analysed. In 10 GC studies, seven tRFs were considerably upregulated and five tRFs were significantly downregulated when compared to controls. Risk of bias was rated low for index test, and flow as well as timing domains in relation to the review question. The applicability of the index test, flow and timing and patient selection for 10 studies was deemed low. In this study, we review the advances in the study of tRFs in GC and describe their functions in gene expression regulation, such as suppression of translation, cell differentiation, proliferation and the related signal transduction pathways associated with them. Our findings may offer researchers new ideas for cancer treatment as well as potential biomarkers for further research in GC.

## 1 INTRODUCTION

**FIGURE 1:**
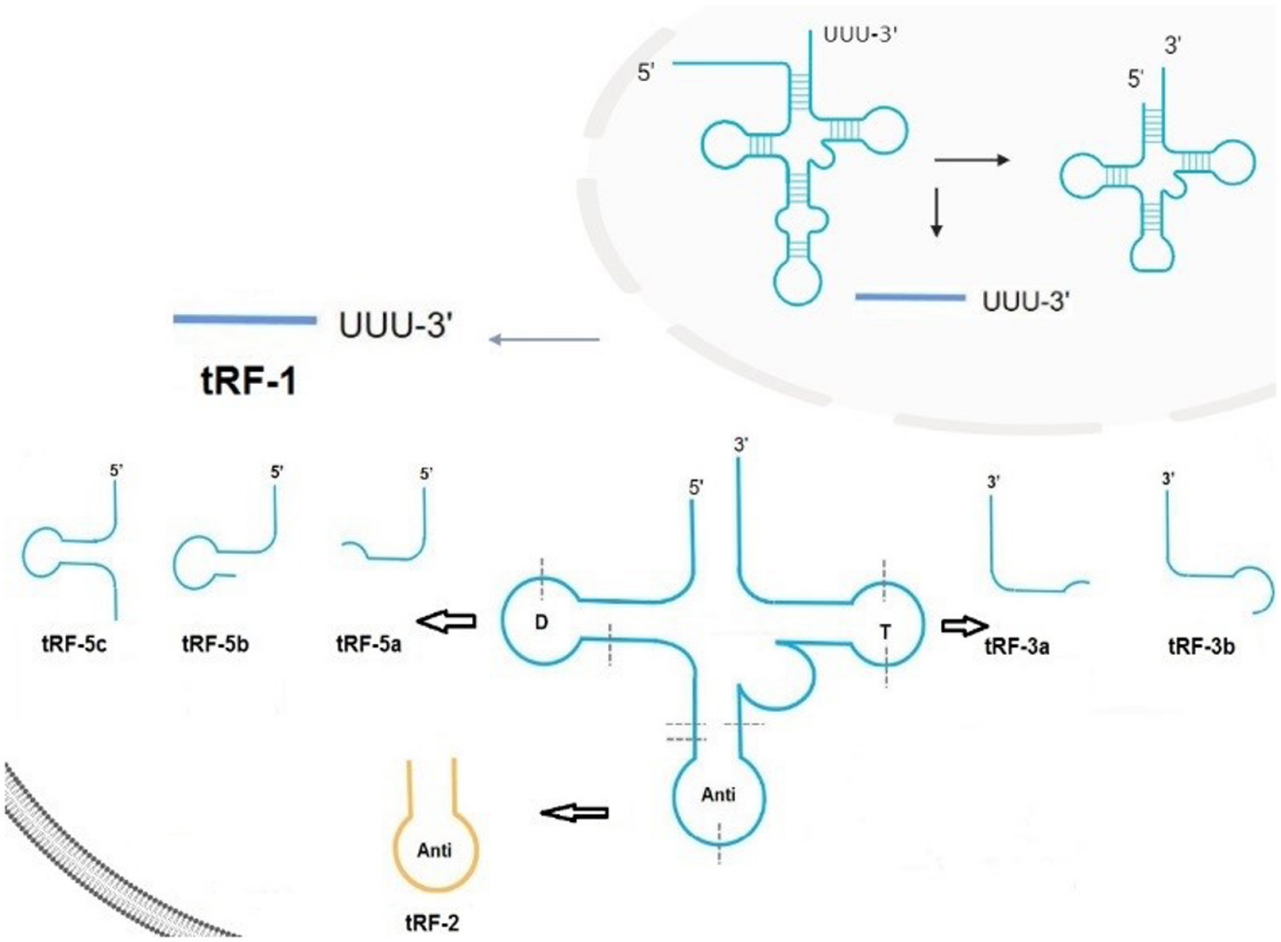
The classification of tRFs. tRF‐1, ‐2, ‐3 tRF‐5 are examples of tRNA‐Derived Fragments (TRFs). tRF‐1 is generated from the 3′‐end of pre‐tRNA. tRF‐2 is a tRNA fragment with an anti‐codon loop generated by an unknown cleavage method. tRF‐5 and tRF‐3 are derived from 5′‐, 3′‐ the ends of mature tRNAs, respectively. i‐tRF is derived from the mature tRNA's internal region.

As the fourth most prevalent tumour of the digestive system and the 3rd most common cause of tumour‐associated death, gastric cancer (GC) continues to rank highly among gastrointestinal cancers, with over 951,000 new morbidities identified every year, corresponding to more than 723,000 fatalities, according to epidemiological surveys.^1^ It is well known that GC occurs in a wide range of geographical areas, including Eastern Europe, sections of Central and South America and East Asia (Japan, Korea, China).^2^ Eastern Europe has the highest rate of GC in Europe, with 70,000 cases per year (Belarus area).^3^ Australia, North America, New Zealand, Southern Asia and North and East Africa have lower rates (10 per 100,000 in men).^4^ Adjuvant chemoradiotherapy, immunotherapy, neoadjuvant chemoradiotherapy and surgery are being used to treat GCs, but treatment results are restricted in advanced‐stage patients. Surgical resection can effectively control early‐stage GC.^5^ However, GC is linked to a significant risk of metastasis and resistance to traditional treatments like chemotherapy and radiotherapy, resulting in a poor prognosis.^6^ The gastric cancer‐related high mortality rate is primarily attributed to delayed diagnosis as a result of the lack of screening programs: the scarcity of particular early clinical signs and the prevalent kinds of recurrence, such as hematogenous spread, peritoneal dissemination and lymph node metastases, as well as the dearth of desirable molecular biomarkers.^7^ A poor prognosis is largely due to an insufficient understanding of genetic diversity and molecular mechanisms of GC incidence and development. As a result, recognition of high‐risk patients and early detection of GCs with a poor prognosis is critical.^8^ tRFs have recently attracted a lot of attention as researchers try to figure out what role they play in disorders. tRFs, a new class of short non‐coding RNAs formed from tRNA mature sequences or precursors, are involved in the advancement of cancer and carcinogenesis.^9^ The lengths of tRFs are about 14–30 nucleotides (nt), Among them, 5′tRF has 5′‐Cleavage of precursor and mature tRNAs at various sites yields these tRFs. tRNA‐derived ncRNAs can be assigned into two categories: tRFs (tRNA‐derived fragments) and tiRNAs (or tRNA halves). tiRNAs are made from particular cleavage in mature tRNA's anticodon loops while tRFs are made from precursor or mature tRNA.^10^ tRFs/tiRNAs are grouped into five categories based on their mapped sites on the mature or precursor tRNA transcript: tiRNA and tRF‐1, ‐2, ‐5, tRF‐3 (Figure 1). Among them, 3′tRF has 3′‐hydroxyl while 5′tRF has 5′‐phosphate groups, these tRFs inhibit mRNA translation by combining 5′ or 3′ ends with a conservative region of 3′‐UTRs in mRNAs. According to their biological roles, tiRNAs/tRFs can be assigned into three categories: epigenetic regulation, RNA silencing and translation regulation.^11^

In recent years, cancer research using tiRNAs/tRFs has focused on these three areas. Human diseases, including metabolic diseases and disorders, cancer, have been linked to abnormal expression of tRFs/tiRNAs.

tRFs regulate cancer progression via competitive binding of RNA binding proteins.^12^, ^13^, ^14^ Moreover, tRFs have RNA silencing roles that are comparable to those of miRNA, which involves the formation of RNA‐induced silencing complex (RISC) with Argonaute (AGO) protein to silence mRNA.^15^, ^16^ A particular tRNA‐derived small RNA (tsRNA) referred to as LeuCAG 3′ tsRNA binds ribosomal protein mRNAs for efficient translation.^17^ Some tsRNAs exhibit the same sequence as miRNAs, with comparable mechanisms of action.^18^ TsRNAs sequences from tRNALeu, tRNALys, and tRNAAla are comparable to those of miR‐1274a/b, miR‐1280, and miR‐886‐5p, respectively.^19^ Haussecker et al. reported that various tsRNAs regulate RNA silencing to control genes via differential AGO protein linkage.^18^ Yamasaki et al. documented that after transfection of 5′tiRNA into the human osteoblast cell line U2OS, translation was suppressed due to the presence of the oligoguanine motif at its end.^20^ Therefore, tRFs are involved in regulation of protein translation, gene expressions as well as several cellular activities.^21^

Differentially expressed tRFs have been reported in GC clinical samples.^22^ Thus, tRFs have vital roles in cancer pathogenesis. Tong et al. reported abnormally expressed tRF‐3017A (a derivative of tRNAVal‐TAC) in GC tissues as well as cell lines and verified its effect on enhancing GC cell invasion and migration. tRF‐3017A, a mature tRNA‐Val‐TAC 3′ end degradation product, is made up of 19 nt. Therefore, it was postulated that tRF‐3017A are abnormally elevated in GC lymph node metastasis patients.^23^ tRF‐3017A promotes GC cell proliferation via targeting and regulating NELL2, its downstream mRNA. Nerve epidermal growth factor‐like like protein (NELL), which was initially found in chicken as a polymeric and multimodular extracellular glycoprotein.^24^ Two mammalian NELL homologues (NELL1 and NELL2) have been established in human foetal brain cDNA library.^25^ NELL2, which was found to be abundant in the nervous system, has roles in neural development.^26^, ^27^, ^28^ Relative to nervous system tumours, NELL2 is abundant in normal nerve cells.^26^

Zhu et al. reported that tRF‐5026a suppressed GC cell proliferation, migration, and cycle progression by regulating the PTEN/PI3K/AKT signalling pathway,^29^ which has roles in various cell functions, including differentiation, growth, proliferation, invasion motility, and intracellular trafficking. The PTEN gene on chromosome 10q23.3 inhibits tumour growth by suppressing PI3K pathway activation.^30^

Gu et al. reported that hsa_tsr016141 is a potential tumour marker. Relative to normal controls, serum and tissue hsa_tsr016141 expressions in GC patients were elevated. These expressions increased with increasing lymph node metastasis as well as tumour grade, implying a good diagnostic efficacy for GC patients.^31^

Shen et al. reported markedly low plasma tRF‐33‐P4R8YP9LON4VDP levels in GC patients, relative to healthy individuals. Moreover, tRF‐33‐ P4R8YP9LON4VDP suppressed GC cell proliferation and was established to be a potential tumour biomarkers.^11^

Shen et al. found that tRF‐19‐3L7L73JD suppresses cell proliferation as well as migration, induces apoptosis, and arrests cells at the G0/G1 phases, implying inhibitory roles in GC development.^32^

Dong et al. discovered that levels of tRF‐24‐V29K9UV3IU and its target genes (CCND2, FZD3, as well as VANGL1) in GC cells and tissues were suppressed, implying that tRF‐24‐V29K9UV3IU inhibits GC progression by suppressing cell migration, proliferation, as well as invasion, while enhancing cell apoptosis via regulation of the Wnt pathway.^33^

Huang et al. reported varying serum tRF‐31‐U5YKFN8DYDZDD levels in para‐cancerous and tumour tissues from GC patients and healthy people as well as from GC patients prior to and after surgery. Thus, tRF‐31‐U5YKFN8DYDZDD is a diagnostic and a prognostic marker for poor outcomes in GC patients.^34^

In another study, Lin et al. found elevated plasma exosomal tRF‐25, tRF‐18, tRF‐38 levels in GC, relative to controls, implying a potential diagnostic role in GC.^35^

Xu et al. established markedly low tRF‐Glu‐TTC‐027 levels in GC samples, which was also shown to regulate the MAPK pathway. Thus, as a tumour suppressor, tRF‐Glu‐TTC‐027 is a potential treatment target for GC.^36^

**FIGURE 2:**
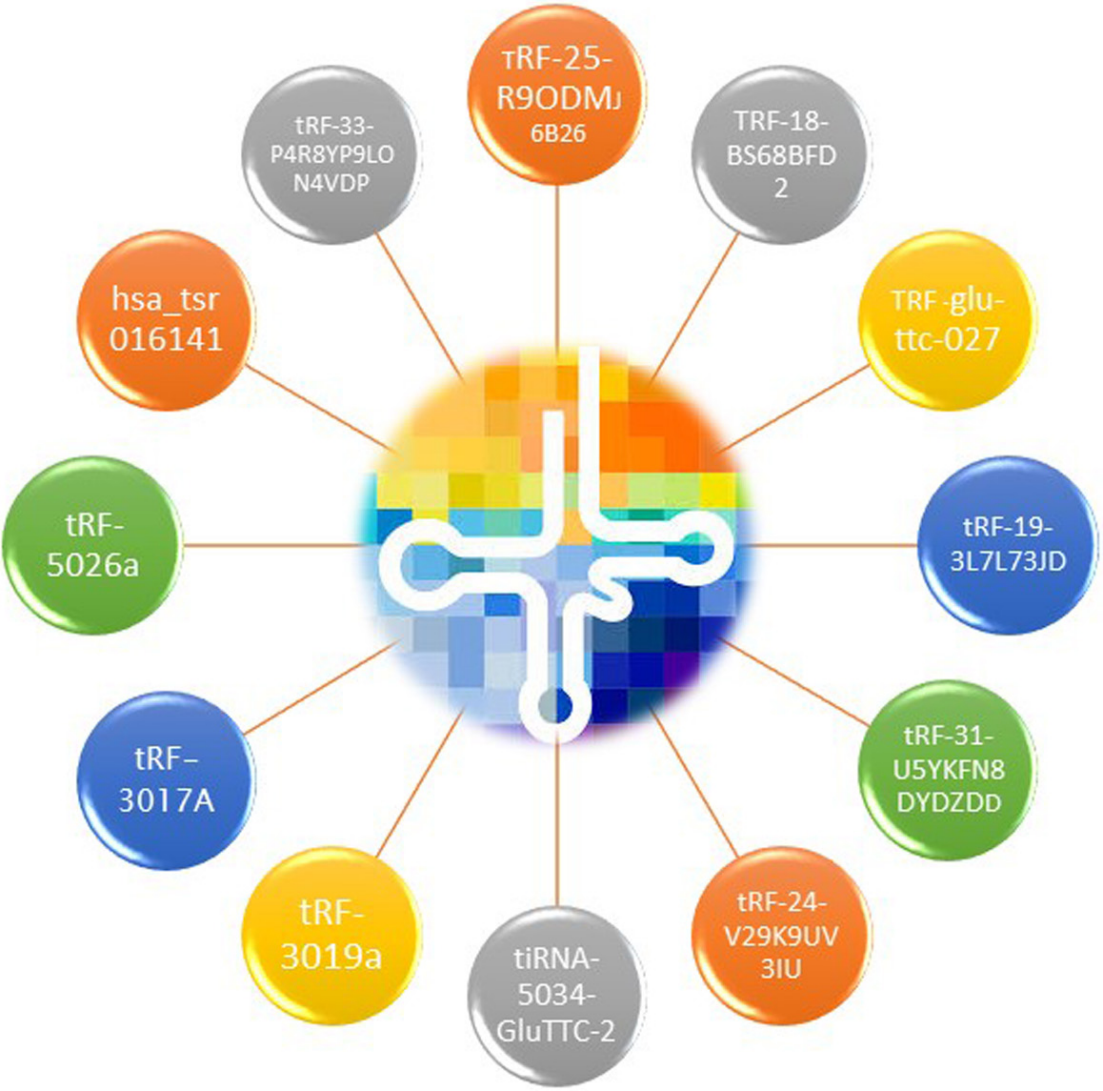
tRFs used as diagnosis biomarkers. We discovered that multiple different tRFs from both the up‐ and down‐regulated tRFs groups were involved in GC. Some tRFs were upregulated and others were downregulated.

Zhang et al. found that tRF‐3019a‐induced FBXO47 suppression enhances GC cell malignant activities, implying that tRF‐3019a has vital roles in GC by targeting FBXO47. Thus, tRF‐273019a regulates GC cell migration, proliferation as well as invasion via targeting FBXO47, and maybe a diagnostic marker^37^ (Figure 2).

## 2 METHODS

### 2.1 Search strategy

Scopus, EMBASE and PubMed/Medline were extensively searched up to the 15th of February 2022. In our literature search, three keywords were applied: “GC tRFs”, “tRNA‐derived fragments”, and “GC”. In addition, the reference lists of approved publications were carefully examined for supplemental sources.

### 2.2 Selection criteria

The following criteria were used to determine inclusion:

1. Studies needed to report dysregulated tRFs in GC patients, as well as tRF profiling with GC patients.
2. Using this method to determine levels of tRFs in GC serum or plasma and tissue, the study was conducted with QRT‐PCR.

The following was the list of exclusion criteria:

1. Studies with limited or incomplete data.
2. Non‐human and non‐English subject researches.
3. Abstracts, reviews, comments, and letters.

### 2.3 Quality assessment and data extraction

The first author's name, sample size, tRFs profiles, specimen source, publication year, expression levels were all collected from the included literature. Two investigators (Kohansal and Ghanbariasad) assessed the quality of enrolled studies using the Quality Assessment of Diagnostic Accuracy Studies‐2 (QUADAS‐2) method, which is based on 4 dimensions (“Patient Selection,” “Index Test,” “Reference Standard,” as well as “Flow and Timing”) in 2 groups (“Applicability Concerns” and “Risk of Bias”).^17^ Following a debate, any differences were settled by consensus.

## 3 RESULTS

**FIGURE 3:**
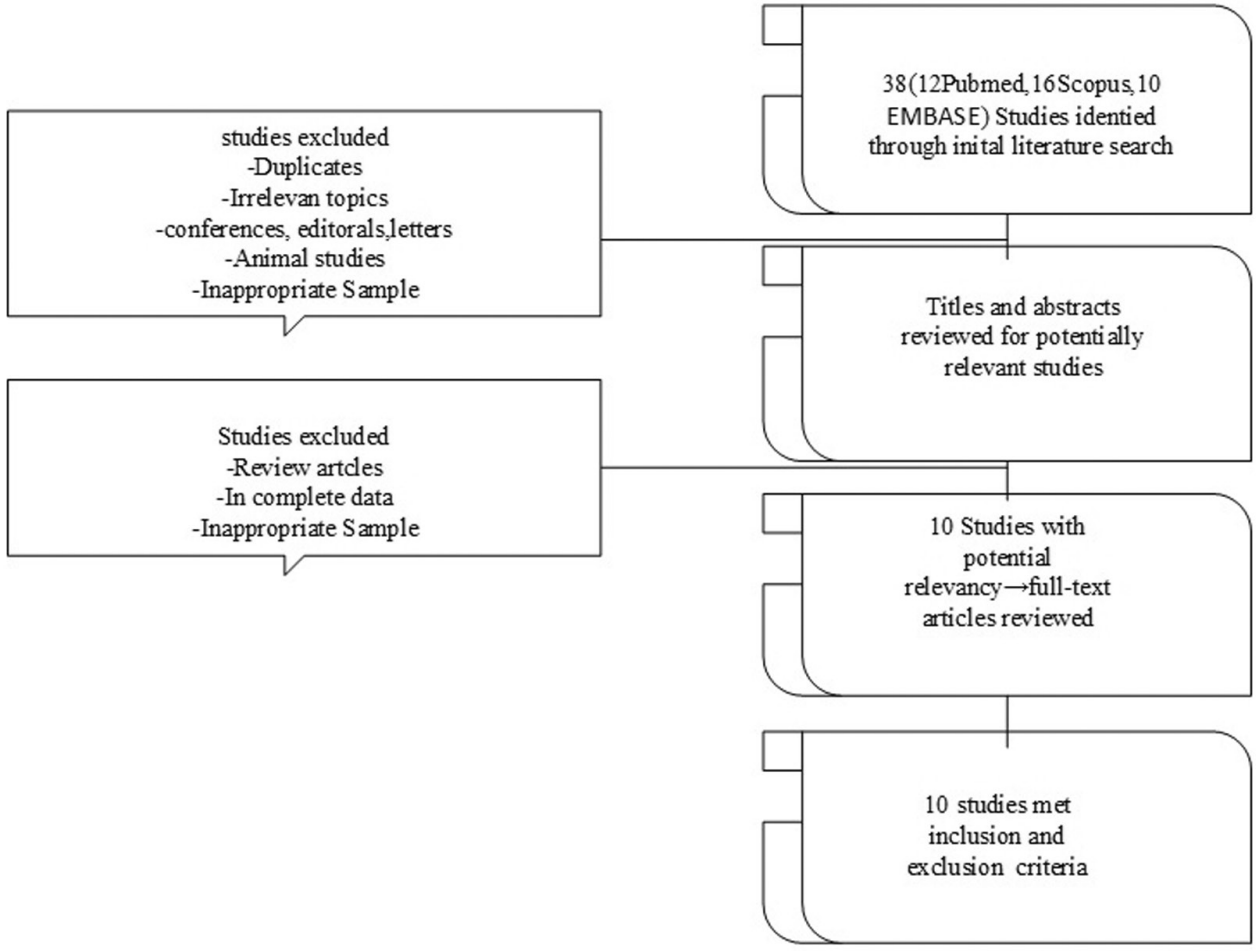
The study selection process is depicted as a flow diagram. A flow diagram of the study selection process was showed. A total of 38 studies in PubMed/Medline, EMBASE and Scopus was retrieved. All studies were screened according to role of tRFs in GC. A final total of 10 articles, including tRFs studies for comparing the expression of tRFs in GC patients and healthy people were used for the paper review.

Using our search method, a total of 38 studies in PubMed/Medline, EMBASE and Scopus was retrieved. All studies were screened according to role of tRFs in GC. A final total of 10 articles, including tRFs studies for comparing the levels of tRFs in GC patients and normal controls were used for the paper review. Figure 3 shows the selection process for this study.

### 3.1 Quality assessment

Because all studies used a case–control design and patients were not enrolled consecutively or randomly, the bias risk was high in patient selection domains and unclear for the reference standard domains using the QUADAS‐2 instrument. Furthermore, it is unclear how findings of the reference standard may be understood without establishing the index test results. The tRFs expression levels of all enrolled patients were compared with a normal control group.

**FIGURE 4:**
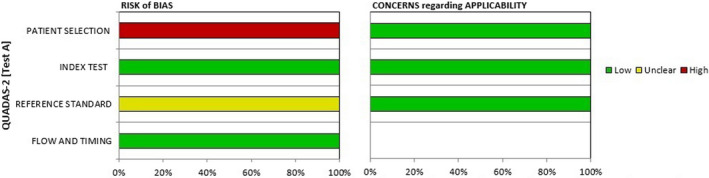
Methodological quality graph. The comprehensive results of the QUADAS2 quality evaluation were showed. These studies did not provide sensitivity and specificity data.

Risk of bias was rated low for the flow, timing and index test domains in relation to the review question. The applicability of the index test, flow and timing and patient selection for ten studies was considered low. Finally, Figure 4 shows the comprehensive results of the QUADAS2 quality evaluation. These studies did not provide sensitivity and specificity data.

### 3.2 Study characteristics

A total of 928 GC patients were studied in ten investigations. tRFs' utility in distinguishing GC patients from normal controls. The earliest study was published in 2020, and the majority of them were published last year (2021). All these studies were completed in Chinese population. The number of GC patients investigated in each study ranged from 33 to 193. Did not have a known history of other medical conditions that could potentially interfere with their performance GC patients. Among the 10 studies, tRFs were extracted from tissue (four studies), both serum and tissue (three studies), plasma (two studies) and one study examined tRFs extracted from blood exosomes.

All studies examined pre‐selected candidate tRFs using real‐time quantitative PCR, and the number of tRFs examined in each study ranged from one to five. Seven studies^11^, ^29^, ^33^, ^34^, ^35^, ^36^, ^37^ used a genome‐wide discovery approach with high throughput sequencing or small RNA sequencing to identify candidate tRFs, which were then validated in an independent cohort using qRT‐PCR. Using this approach, three of these three studies^11^, ^32^, ^33^ selected a panel of tRFs to distinguish GC from normal controls. Table 1 summarizes the study's characteristics.

**TABLE 1:** Main Characteristics of tRFs used as potential biomarkers of GC

tRF name/ID	Study	Region	Expression level	Test method	Sample type	Sample size	Biological effect	Ref
tRF‐3019a	Zhang 2020	China	Upregulated	qRT‐PCR	Tissue	112	Regulates tumour suppressor 26 gene FBXO47	^23^
tRF‐Glu‐TTC‐027	Xu 2021	China	Downregulated	qRT‐PCR	Tissue	33	Tumour suppressor	^36^
tRF‐33‐P4R8YP9LON4VDP	Shen 2021	China	Downregulated	qRT‐PCR	Blood	89	Tumour suppressor	^11^
tRF‐31‐U5YKFN8DYDZDD	Shen 2021	China	Upregulated	qRT‐PCR	Tissue Serum	111	Promotion of cell division and regulation of transcription	^34^
tRF‐19‐3L7L73JD	Shen 2021	China	Upregulated	qRT‐PCR	Plasma	129^a^	Tumour suppressor	^32^
hsa_tsr016141	Gu 2021	China	Downregulated	qRT‐PCR	Tissues Serum	193^b^	RNA silencing by binding to downstream mRNA	^31^
tRF‐5026a	Zhu 2021	China	Downregulated	qRT‐PCR	Tissue Plasma	86	Suppression of proliferation, migration by regulating the PTEN/PI3K/AKT	^29^
tRF‐38‐QB1MK8YUBS68BFD2 tRF‐18‐BS68BFD2 tRF‐25‐R9ODMJ6B26	Lin 2020	China	Upregulated	qRT‐PCR	Plasma (exosome)	50	Differential expression between patients and controls	^35^
tRF‐3017A	Tong 2021	China	Upregulated	qRT‐PCR	Tissue	87	Regulates the tumour suppressor gene NELL2 through forming the RISC with AGO	^23^
tRF‐24V29K9UV3IU	Dong 2020	China	Downregulated	qRT‐PCR	Tissue	38	Inhibiting cell proliferation, while promoting cell apoptosis by regulating the Wnt	^33^

^a^ Validation group contained 89 matched plasma samples from GC patients a day prior and 7 days post‐surgery. The test cohort had 40 plasma samples from GC patients.

^b^ The study involved 130 cases of GC and 63 post‐operative samples after the operation from GC patients.

### 3.3 Dysregulated tRFs in GC

Dysregulated tRFs that were found to have different expression levels between GC patients and normal controls are shown in Table 1. Among differentially expressed tRFs, the upregulation of tRF‐3019a, tRF‐31‐U5YKFN8DYDZDD, tRF‐38‐QB1MK8YUBS68BFD2, tRF‐19‐3L7L73JD, tRF‐3017A, tRF‐18‐BS68BFD2, tRF‐25‐R9ODMJ6B26, and downregulation of tRF‐24‐V29K9UV3IU, tRF‐5026a, hsa_tsr016141, tRF‐33‐P4R8YP9LON4VDP, tRF‐Glu‐TTC‐027 were reported in five and five studies, respectively.

## 4 DISCUSSION

With the highest death rate, GC is a prevalent global malignant tumour. Early‐stage GC patients usually have no discomfort symptoms. Subsequently, the majority of them are diagnosed in late or middle stages of disease.^6^ Advanced‐stage GC patients have a 5‐year post‐operative survival rate of as low as 25 percent. Nevertheless, for early‐stage GC, the 5‐year post‐operative survival rate is 90–95 percent. As a result, early‐stage GC has a significantly high 5‐year survival rate than advanced‐stage GC.^38^ Today's tumour markers for GC have low specificity as well as sensitivity, and by the time GC is discovered, it is in advanced stages.^39^ Consequently, finding highly specific and sensitive markers suitable for early GC diagnosis is critical. It was in the late 1970s that tsRNAs were discovered. Thus, it was first thought to be the result of random tRNA degradation, and it received little attention.^40^ tsRNAs, a type of small non‐coding RNA (sncRNA), could be used in liquid biopsy because of their abundance in body fluids and their stable structure, as well as they could be the start of a new generation of tumour biomarkers. tsRNAs have been suggested as proposed biomarkers in ovarian cancer,^47^ renal cell carcinoma,^41^, ^42^ breast cancer,^43^, ^44^ colorectal cancer,^9^, ^45^ lung cancer,^46^ prostate cancer^48^, ^49^ and others.^50^, ^51^ For example, current research has indicated that considerably changed tRFs (5′ tRNA halves) identified in BC serum are strongly linked to subtypes of the BC and clinicopathologic characteristics, whereas miRNA‐like tRFs are highly linked to tumour growth in GC.^52^

**FIGURE 5:**
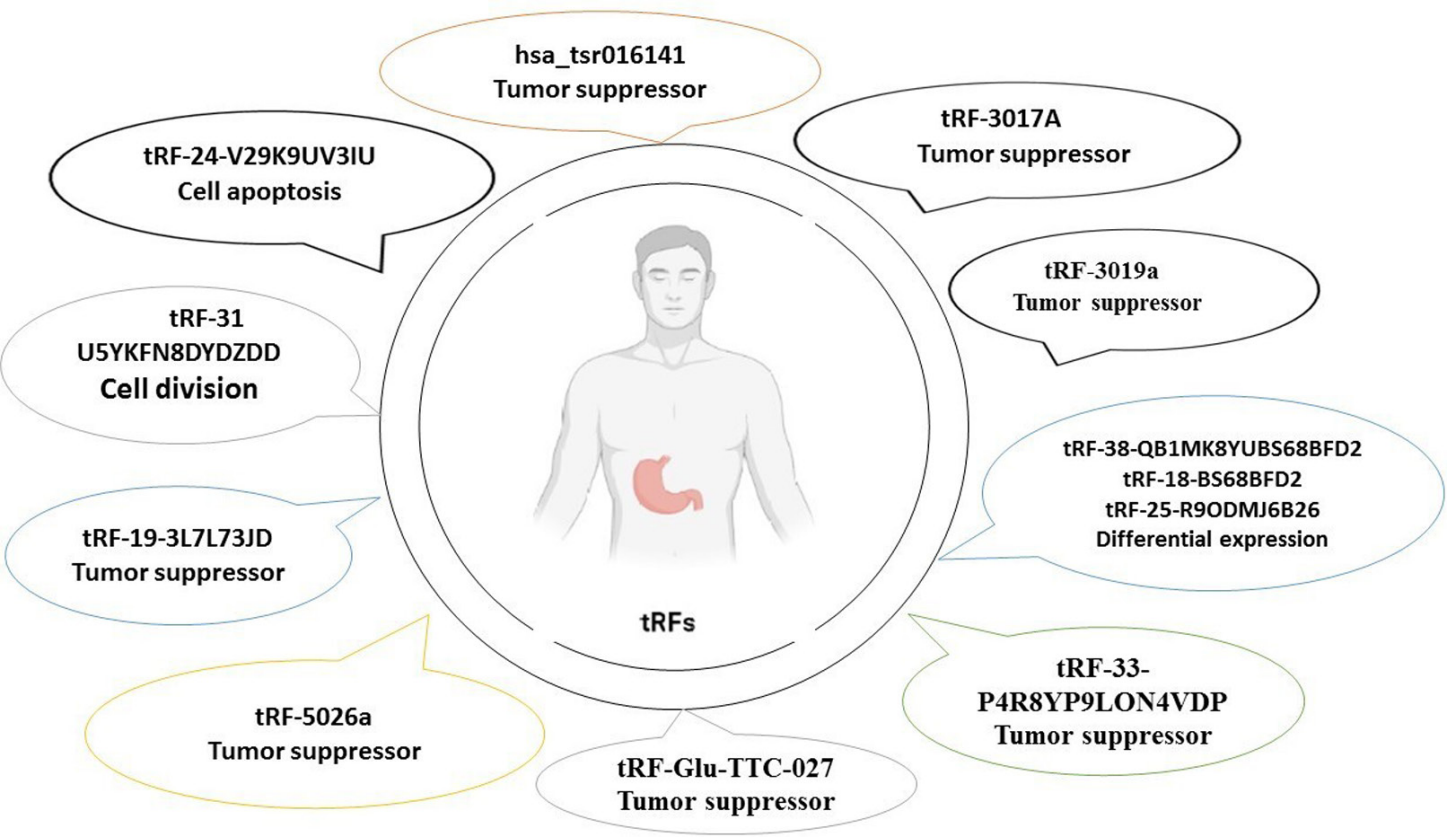
Characteristics of tRFs used as potential biomarkers of GC. The significance and characteristics of multiple tRFs in gastric tumour development and therapeutics.

This study was obtained through a review of 10 articles. These studies support the hypothesis that tRFs may be useful biomarkers for GC. In light of the newly published studies, we gain a better understanding of the current scenario and the prognosis of GC patients. Hence, we chose studies published until February 2022. This is the first review summarizing the discoveries and current research on tRFs in GC. We hypothesize that tRFs may be used in the prognosis of GC patients. We discovered that 12 different tRFs from both the up‐ and down‐regulated tRFs groups were involved in GC. Seven tRFs were upregulated and 5 tRFs were downregulated (Figure 5).

The first report of the significance of tRFs in gastric tumour development was presented by Shen's group in 2021,^32^ who indicated that tRF‐19‐3L7L73JD, affected cell cycle and arresting cells at G0/G1 phases. The investigators discovered a link between increased tRF‐19‐3L7L73JD levels and the ability of GC cells to migrate. Relative to the negative control (NC), cell proliferations were suppressed by upregulated tRF‐19‐3L7L73JD. This tRF promoted GC cell apoptosis and inhibited their migration as well as proliferation. Furthermore, the expression of tRF‐19‐3L7L73JD in patients was associated with tumour size which has a significant impact on patient prognosis. The area under receiver operating characteristic (ROC) curve of tRF‐19‐3L7L73JD is 0.6230. The specificity and sensitivity are 0.7959 and 0.4045, respectively, implying a diagnostic value of tRF‐19‐3L7L73JD. Due to several changes, tsRNAs are more stable in plasma than other sncRNAs.^53^ These findings imply that tRF‐19‐3L7L73JD, which plays a suppressive role in GC, could open up new avenues for molecular therapeutics in GC diagnosis as well as treatment. tRFs have regulatory effects in tumour progression. Slimily, in breast cancer (BC), various tsRNAs are aberrantly expressed and are potential diagnostic and prognostic markers. Honda et al. reported upregulated SHOT‐RNA in androgen receptor (AR)‐positive prostate tumour and oestrogen receptor (ER)‐positive breast tumour cell lines.^54^ Plasma tRF‐Arg‐CCT017, tRF‐Gly‐CCC‐001 and tiRNA‐Phe‐GAA‐003 levels in BC patients were markedly elevated and were associated with OS and disease‐free survival (DFS).^55^ In triple‐negative BC (TNBC) patients, tRF‐31‐87R8WP9I1EWJ0 levels was suppressed.^56^ Nevertheless, in non‐TNBC patients, tRF‐18‐18VBY9DV and tRF23‐NB57BK87DZ levels from tRNAGlyCCC‐5‐1 and tRNAPheGAA‐2‐1 were markedly suppressed.^57^ Thus, aberrant tRF levels correlate with alterations in tumour function and are viable diagnostic or prognostic markers or even treatment targets. The tRNA‐Derived Fragment tRF‐24‐V29K9UV3IU functions as a miRNA‐like RNA to inhibit GC progression by suppressing GPR78.

Currently, Gu et al. (2021) revealed that hsa_tsr016141, which was markedly elevated in GC patients, is a potential GC marker.^31^ The hsa_tsr016141 levels increased with increasing tumour grade and lymph node metastasis. Relative to normal controls, hsa_tsr016141 levels in GC patients were associated with tumour grade and lymph node metastases, indicating that it had strong diagnostic efficacy in GC patients. ROC, sensitivity and specificity analysis showed that the serum expression level of hsa_tsr016141 could significantly distinguish GC patients from healthy donors or gastritis patients. After GC surgery, serum hsa_tsr016141 levels were established to be markedly suppressed in the same patient. Survival rates for the low expression group were higher, relative to those of high expression group, implying that hsa_tsr016141 can track post‐operative outcomes for GC. Authors compared hsa tsr016141 levels between two groups, Helicobacter pylori‐negative and positive patients, during this event, changes in hsa tsr016141 levels among patients with gastritis who were Helicobacter pylori‐negative or positive were insignificant, indicating the absence of important associations between Helicobacter pylori and hsa tsr016141 levels. These findings show that hsa tsr016141 has a good specificity and stability for clinical uses, with a maximum diagnostic efficiency. Hsa tsr016141 also may effectively track post‐operative conditions of GC and serve as a dynamic monitoring tool in these patients. Some studies have indicated that 3′tRF and 5′tRF are affected by RNA silencing, whereas others claim 5′tRF can block protein translation and represents a new gene regulatory approach.^58^ tsRNAs have been shown to suppress translation.^59^, ^60^ 3′tRF from tRNALeu‐CAG of non‐small cell lung cancer (NSCLC) cells exhibits comparable effects to those of miRNA and can ameliorate protein translation.^35^, ^59^ 5′ tiRNACys and 5′tiRNAAla could suppress translation by establishing intermolecular RNA G quadruplexes (RG4) to substitute for eIF4G/eIF4E, a translation initiation complex located on the mRNA cap.^36^ The regulation of hsa tsr016141 which belongs to the 5′tRF and is located in the nucleus.^61^ May have an impact on RNA silencing or protein translation through binding to downstream mRNA. To summarize, hsa tsr016141 has the potential to diagnose GC early and monitor it after surgery. The tRFs have potential clinical uses in cancer diagnosis.^11^, ^62^, ^63^ For example, in colorectal cancer cells, tRF‐24‐NMEH623K25, tRF‐29‐QU7BPN6ISB JO, tRF‐30‐XSXMSL73VL4Y and tRF‐27‐Q99P9P9NH5N levels were markedly elevated significantly and markedly correlated with tumour cell differentiation.^64^ These tRFs are possible diagnostic markers for colorectal cancer. Huang et al. reported that tRF/miR‐1280 from tRNALeu and pre‐miRNA was suppressed in colorectal cancer tissues.^9^ Overexpressed tRF/miR‐1280 suppressed Notch1 as well as Notch2 receptors and downregulated cell proliferation as well as colony formation to reduce tumour growth and metastasis.

Tong et al. (2020) found that tRF‐3017A inhibited NELL2, a tumour suppressor, at two levels: in vitro and in vivo.^23^ tRF‐3017A, a degradation product for mature tRNA‐Val‐TAC that is specifically cleave at the 3′ end of T‐loop. Levels of tRF‐3017A in 71.2% pairs of GC tissue were established to be upregulated, relative to matched‐paired non‐cancerous adjacent tissues (NATs). Elevated tRF‐3017A levels enhanced GC cell migration as well as invasive abilities, which were suppressed by inhibited tRF‐3017A levels. Therefore, elevated tRF‐3017A levels correlated with lymph node metastasis. GC metastasis highly affects prognosis. Using qRT‐PCR, expressions of NELL2 compared with tRF‐3017A. mRNA levels for NELL2 were suppressed in tumour tissues, relative to NATs and GC tissues. NELL2 and tRF‐3017A levels have a moderate negative connection, according to correlation analysis. Overexpression and knockdown experiments confirmed GC cell migration as well as invasion could be inhibited by NELL2. tRF‐3017A which promotes invasion and migration, inhibits NELL2 through a mechanism involving RISC with AGO proteins in GC. The complement of tRFs and target mRNA plays a critical role in RNA silencing according to some studies. It was discovered that tRFs bind to Ago2, is important during RNA interference, in order to recognize targets.^16^, ^65^, ^66^ A complex of tRF‐3017A and Ago2 is possible for silencing NELL2 which is downstream of tRF‐3017A, according to luciferase report analysis and (RNA immunoprecipitation) RIP‐Ago2 assay results. Therefore, tRF‐3017A precisely binds the 3′ UTR of NELL2 via interactions with Ago2 and negatively regulate NELL2 expressions. There has been evidence that NELL2 is abundant in normal nerve cells, relative to nervous system tumours, and it blocks the proliferation in renal cancer. In addition, NELL2 also is enriched in para‐cancer cells and inhibits the growth of clear cells.^26^, ^67^ In conclusion, these findings reveal that tRF‐3017A enhances GC cell migration as well as invasion. In addition, tRF‐3017A enhances GC by regulating NELL2 via miRNA‐mediated target gene silencing. tRFs have vital roles in oncogenesis and tumour development.^63^, ^68^, ^69^ tRF‐1001 is vital in colorectal cancer growth, and its knockdown arrests cancer cells in the G2 phase.^70^ Stably expressed tRF‐CU1276 suppresses a DNA dynamics regulator (RPA1), thereby regulating damage responses to molecular and suppressing lymphoma cell proliferation.^19^ The novel tRF‐1280, formerly referred to as miR‐1280, has the ability to suppress colorectal cancer metastasis.^9^ In addition, BC metastasis as well as progression can be suppressed by endogenous tRFs substituting YBX1.^13^

In GC, Shen et al. (2021) revealed that tRF‐33‐P4R8YP9LON4VDP, as a tRF, is involved in cell proliferation stimulation and apoptosis inhibition.^11^ Relative to plasma samples from GC patients 7 days post‐surgery and healthy controls, qRT‐PCR revealed suppressed plasma tRF‐33‐ P4R8YP9LON4VDP levels in GC patients at one day prior to surgery. Thus, tRF‐33‐P4R8YP9LON4VDP may have an inhibitory role in **GC**. In ovarian cancer, tRFleu‐CAG regulates downstream target genes.^71^ *Aurka* levels were suppressed when tRFLeu‐CAG was inhibited. In high‐grade serous ovarian cancer, tRF‐03357 downregulated HMBOX1, a hepatocyte nuclear factor family member.^71^ HMBOX1 has various roles in occurrence of several tumours. Elevated HMBOX1 in GC leads to poor prognostic outcomes by enhancing cell proliferation as well as migration.^72^ The influence of tRF‐33‐P4R8YP9LON4VDP downregulation or overexpression in GC cell apoptosis and cell cycle as well as migration, proliferation were investigated to determine the basis of this tRF's mechanical action. Colony formation assays confirmed that transfected with tRF‐33‐P4R8YP9LON4VDP inhibitors increased proliferation whereas, transfection with tRF‐33 P4R8YP9LON4VDP mimics suppressed GC cell proliferation. Furthermore, upregulated tRF‐33‐P4R8YP9LON4VDP enhanced the distribution of cells in G1 phase, implying its ability to arrest cells at G0/G1 while the authors disclosed, inhibition of tRF‐33‐P4R8YP9LON4VDP led to more cells were arrested at G2/M phase. As a result, these outcomes, tRF‐33‐P4R8YP9LON4VDP plays essential role in GC cell invasion and progression, and maybe a therapeutic target and potential new prognostic biomarker for GC cancers.

The involvement of tRF‐31 U5YKFN8DYDZDD in triggering the GC cell, regulation of transcription, cell division and signal transduction have recently been discovered by Huang (2021).^34^ GC patients have markedly elevated tRF‐31‐U5YKFN8DYDZDD levels, relative to healthy donors, implying that it has a lot of potential as a new “liquid biopsy” diagnostic for GC. tRF‐31‐U5YKFN8DYDZDD levels in serum, tumour tissue, and cell lines of GC was found to be much elevated than in normal gastric epithelial cells, para‐cancerous tissues and gastritis patients, general population as well as its expression was linked to tumour invasion, vascular invasion, TNM stage and lymph node metastasis. For instance, elevated tRF‐31‐U5YKFN8DYDZDD levels in GC markedly correlated with various GC stages. tRF‐31‐U5YKFN8DYDZDD exhibited a high sensitivity as well as specificity in differentiation and diagnosis of malignant and benign gastric tumours, relative to conventional biomarkers, including CEA. Combined use of tRF‐31‐U5YKFN8DYDZDD with CA199, CEA and CA724 has a high diagnostic as well as good clinical application potentials. Carbohydrate antigen 199 (CA199), carcinoembryonic antigen (CEA), as well as carbohydrate antigen 724 (CA724) are comparatively mature cancer markers in clinical applications; however, their specificity as well as sensitivity are low.^73^ Yu et al. shows that CEA sensitivity for GC diagnosis is about 13%–35%, and its specificity is 65%, while those of CA199 are 40% and 70%, respectively.^74^, ^75^ Patients with elevated tRF‐31‐U5YKFN8DYDZDD expression had a considerably shorter survival outcomes, compared with low expression patients, indicating that is a prognostic factor. Elevated tRF‐31‐U5YKFN8DYDZDD levels were markedly correlated with short overall survival and its expression was an independent prognostic factor. These findings imply that tRF‐31‐U5YKFN8DYDZDD is a potential marker for GC diagnosis and prognosis. In another research in BC, runt‐related transcription factor 1 (Runx 1) reversed ts‐112‐induced tumour cell proliferation.^76^ tsRNA‐26576 can enhance tumour cell proliferation and enhance BC cell invasion as well as migration.^77^

According to the findings of a recent study, tRF‐24‐V29K9UV3IU, a mitochondrial 5‐tRF, by downregulating the Wnt/β‐catenin signalling pathways^33^ inhibited GC cell invasion, proliferation and migration. Mo et al. reported that 5′‐tiRNA^Val^ suppresses tumours by suppressing the FZD3/Wnt/β‐Catenin pathway. Therefore, tRF has key roles in tumorigenesis and is a potential diagnostic as well as prognostic marker or even a treatment target for tumours.^78^, ^79^ The Wnt pathway facilitates cancer occurrence. As a signalling pathway, Wnt has roles in many important processes during embryonic development, such as organ growth, cell polarization, morphogenesis and migration.^80^ In this regard, the findings of this study revealed that tRF‐24‐V29K9UV3IU which was downregulated in GC, can target several genes connected with traditional signalling pathways that are implicated in tumour occurrence and metastasis, including MAPK (MAPK8IP1, MAPK8IP2, MAPK8IP3, MAP2K3, MAP3K7, etc.), chemokine (CX3CL1, CX3CR1, CXCR3, CXCR5, CXCL9, etc.), Wnt/β‐Catenin (WNT4, WNT10A, WNT3A, etc.), and ErbB (ErbB2) signalling pathways. Furthermore, qRT‐PCR revealed that in GC tissues, tRF‐24‐V29K9UV3IU expressions and those of its target genes (CCND2, FZD3 and VANGL1) were lower than in the control group. These findings revealed that tRF‐24‐V29K9UV3IU by regulating VANGL1, FZD3 and CCND2 played an important function in the Wnt pathway. In addition, tRF‐24‐V29K9UV3IU promotes GC cell apoptosis. In fact, overexpressed tRF‐24‐V29K9UV3IU enhanced cell apoptosis albeit inhibited cell migration, invasion and proliferation in GC. The ROC curve for tRF‐24‐V29K9UV3IU expressions in GC as well as para‐cancer tissues revealed an AUC of 0.8712. The optimal cut‐off value, sensitivity and specificity for tRF‐24‐V29K9UV3IU levels were 1.297%, 78.57% and 92.86% in GC, suggested that tRF‐24‐V29K9UV3IU may be a GC diagnostic marker. To summarize it is believed that has a role in GC development via this signalling pathway as well as may be involved in the metastasis of various tumours. In another study, Wang et al. reported tRF‐24‐V29K9UV3IU suppresses GPR78, thus suppressing GC cell proliferation, migration, invasion and enhancing their apoptosis. In addition, it may act as a miRNA‐like fragment and bind AGO2 to silence the expressions of GRP78 by complementing with 3‐untranslated regions of GPR78 mRNA. Overexpressed tRF‐24‐V29K9UV3IU markedly inhibited MKN‐45 cell proliferation, invasion, migration and their apoptosis while GPR78 ameliorated these effects.^81^ The tRF‐24‐V29K9UV3IU acts as an miRNA‐like RNA to inhibit GC Progression by suppressing GPR78 expressions.

Zhu et al. (2021) in their next study found that tRF‐5026a (tRF‐18‐79MP9P04), which was suppressed in GC, downregulated the migration, proliferation, as well as GC cell cycle progression in vitro and ex vivo via regulation of the PTEN/PI3K/AKT pathway.^29^ Northern blotting it was verified the suppressed tRF‐5026a levels in GC tissues. qRT‐PCR analysis revealed that 79.1% (68/86) of samples had suppressed tRF‐5026a levels. Plasma tRF‐5026a levels in post‐operative patients were elevated, relative to those of pre‐operative patients; that is, after surgical removal of the tumour, plasma tRF‐5026a levels increased to levels of healthy individuals. Mechanistically, elevated tRF‐5026a expressions in both normal and cancerous gastric mucosal epithelial cells caused a G0/G1 block, arrested the cell cycle process, inhibited migration and cell proliferation. Interestingly, in these cells, silencing and suppressing tRF‐5026a expressions induced a block at G2/M, as well as increased proliferation and migration. The diagnostic efficiency of tRF‐5026a as a biomarker in GC, AUC was 0.883, while specificity and sensitivity were 0.676 and 0.973, respectively. After ROC, sensitivity and specificity analysis, tRF‐5026a was shown to have the ability to distinguish GC patients from healthy donors. A survival curve analysis found that tRF‐5026a expression levels in tissues from GC patients correlated with overall survival (OS), where the OS of the low tRF‐5026a expression group was shorter than that of the high expression group. Univariate and multivariate analyses showed that tRF‐5026a was associated with TNM stage and lymph node metastasis and was a good independent prognostic biomarker for GC. The research team found that tRF‐5026a which regulated cell cycle progression, was linked to lymph node metastases, TNM stage and downregulated in GC patients. In addition to its diagnostic value, tRF‐5026a's levels have been associated with tumour size and might predict overall survival in GC, therefore making it a therapeutic target and promising biomarker for GC. Upregulated tRF‐5026a suppressed tumour growth in animal experiments. PTEN/PI3K/AKT signalling pathway is involved in many biological processes, including the cell cycle, cell migration and proliferation. PTEN is a negative regulator of the PTEN/PI3K/AKT signalling pathway, although PI3K and AKT are positive regulators.^82^ PTEN/ PI3K/Akt pathway is related to CSCs in various cancers.^83^, ^84^, ^85^ Dubrovska et al. found that the PTEN/PI3K/Akt pathway was closely related to prostate CSCs and PI3K might be an effective therapeutic target of prostate cancer.^84^ In non‐small cell lung cancer (NSCLC), PI3K/AKT pathway plays a great role in the enrichment of CSCs, thereby promoting the occurrence and development of NSCLC.^85^And some small RNAs such as miR‐873 inhibits the proliferation and differentiation of pancreatic CSCs mediated through PI3K/AKT signalling pathway.^86^ This study reported that increasing tRF‐5026a levels caused an elevation in PTEN levels whereas, a decrease in AKT and PI3K levels in GC cells. On the contrary, decreasing tRF‐5026a, caused lower levels of PTEN and higher levels of PI3K and AKT. PTEN gene encodes a 53 kDa phosphatase protein whose main function is dephosphorylation. This study revealed that tRF‐5026a suppresses GC cell growth via regulation of the PI3K/AKT pathway. Therefore, tRF‐5026a is a potential diagnostic marker for GC.

Recent achieved evidence from an investigation by Xu et al. (2021) has disclosed, through inhibiting MAPK signalling, tRF‐Glu‐TTC‐027 could inhibit GC cell progression.^36^ tRF‐Glu‐TTC‐027 regulated the expressions of MAPK pathway‐related proteins to exert its effects on GC growth.^87^ The MAPK family, such as p38 MAPK, ERK1/2 and JNK, which regulates important cellular processes such as immunity, proliferation, apoptosis and stress responses, may promote the growth of tumours. The three MAPK classical pathways (ERK1/2, JNK, p38) were shown to be regulated by tRFGlu‐TTC‐027.^88^

It is possible that overexpression of tRF‐Glu‐TTC‐027 in vitro and in vivo, inhibited gastric malignancy. On the contrary, tumour size and histological grade are significantly associated with upregulated tRF‐Glu‐TTC‐027 in GC. Various studies reported consistent findings with regards to functions of tsRNAs. tsRNAs are associated with various tumours.^89^, ^90^, ^91^, ^92^ Falconi documented that tRF3E suppresses BC via NCL‐associated mechanisms.^14^ A novel tRFs class influenced YBX1 UTRs via regulation of the stability of various oncogenic transcripts.^13^ In the sum of tRF‐Glu‐TTC‐027 maybe a promising target for GC molecular treatment.

Lin et al. (2020) discovered that the levels of tRF25 (tRF‐25‐R9ODMJ6B26), tRF18 (tRF‐18‐BS68BFD2) and tRF38 (tRF‐38‐QB1MK8YUBS68BFD2) in plasma exosomes from GC patients were considerably greater than those in healthy controls.^35^ Exosomes that originate from multivesicular bodies are membrane vesicles with a diameter of 30 to 150 nanometres. Exosomes are cellular cargo packages containing proteins, RNA, lipids and DNA that have been extracted from a variety of body and cell fluids.^93^ It has also been discovered that tRFs have been detected in exosomes extracted from dendritic cells,^94^ semen, urine,^95^ accounting for more than half of EV‐related RNAs in HEK cells.^96^ The authors developed a model including the three tRFs (tRF‐38, tRF‐25 and tRF‐18) for GC diagnosis with a mean AUC of 0.815. The tRF panel exhibited a high diagnosis sensitivity as well as specificity for GC, relative to each one the three tRFs markers and to some miRNA markers with wide clinical applications.^97^, ^98^ There have also been discovered exosomes isolated from osteoporosis patients' plasma. As mentioned above, the findings described by Lin's study group fit well with those described by Zhang et al (2018). That tRF25, tRF38 and tRF18 were evaluated by qPCR in healthy controls and osteoporosis samples to validate the differential expression.^99^ These results for patients and controls differed on three diagnostic RFs. Therefore, plasma exosomal tRF‐25, tRF‐18 and tRF‐38 could serve as therapeutic targets, new and promising biomarkers.

**FIGURE 6:**
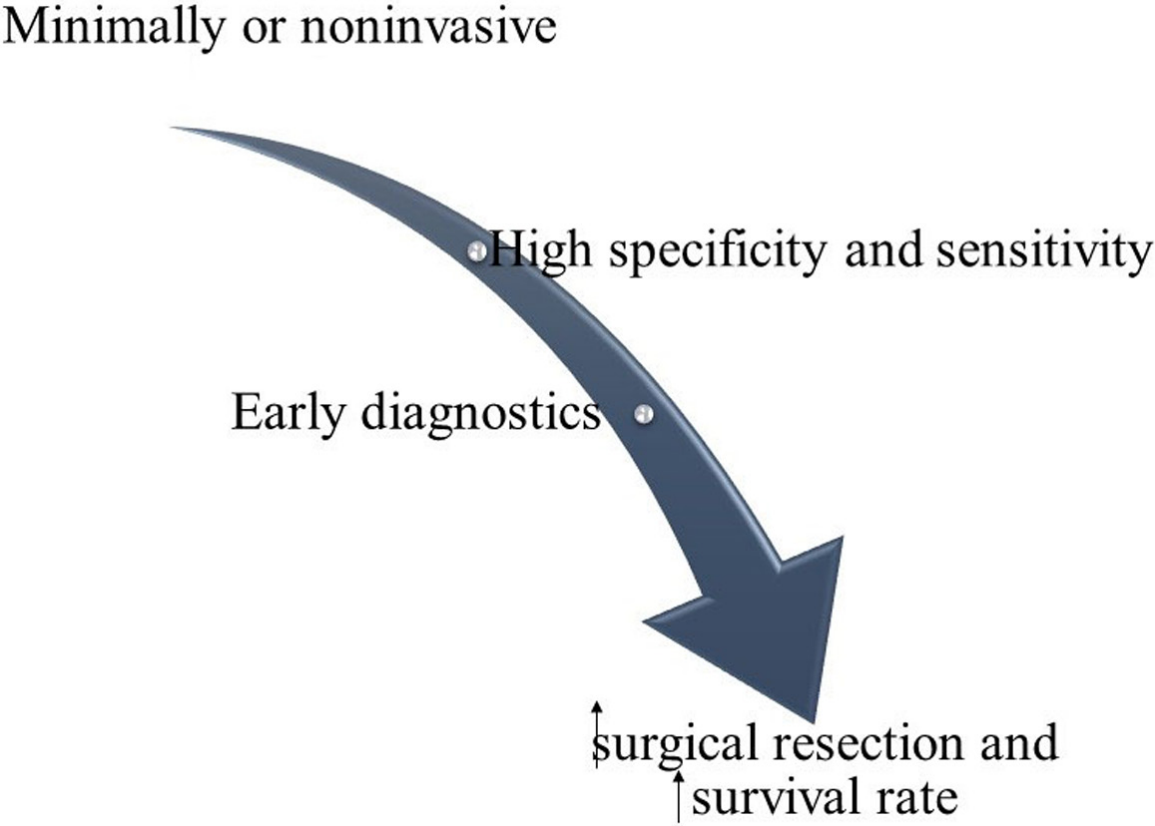
Characteristics required for biomarkers of GC. Biomarkers of GC is minimally or noninvasive, high specificity and sensitivity.

Zhang et al. (2020) indicated that tRF‐3019a which is 73 derived from the 3′ end of mature tRNA‐Ala‐AGC‐1‐1, with cleavage site at the T‐loop, and is located at chr6: 28763741–3 to 75 28763755, induced GC cell invasion, proliferation and migration by targeting F‐box protein 47 (FBXO47) a member of F‐box family and as functions as a 78 tumour suppressor gene. According to the findings, tRF‐3019a upregulates the cancer suppressor gene FBXO47 in GC.^37^ Overexpression of tRF‐3019a, a type of 3′‐tRF, significantly increases proliferation, invasion and migration while the knockdown of tRF‐3019a had opposite effects in GC cells. According to the findings, the luciferase assay confirmed the binding connection between FBXO47 and tRF‐3019a. RNA immunoprecipitation (RIP) assay revealed that FBXO47 and tRF‐3019a form an interaction complex with Ago2. This study determined that tRF‐3019a can bind 3′UTR of FBXO47 to inhibit FBXO47 expressions through interaction with Ago2. Attenuation of FBXO47 expressions partly overturned the effects of tRF‐3019a suppression on GC cell migration, proliferation and invasion. On chromosome 17q12, human FBXO47 operates as a tumour suppressor gene.^100^ tRNA‐derived fragments have been shown to bind mRNA targets to exhibit comparable effects to those of canonical microRNAs.^101^, ^102^ For example, a 3′tRF named CU1276 (tRF‐3027b) binds 3′UTR of RPA1 to suppress its expressions in an Argonaute‐dependent, miRNA‐fashion and regulate responses to DNA damage in B cell lymphoma.^19^ Kuscu et al. documented that 3′tRFs post‐transcriptionally suppresses genes via an Argonaute‐GW182‐containing RISC through sequences that match those of targeted mRNAs.^60^ A 5′fragment of tRNA‐Glu‐CTC (tRF5‐Glu) binds the 3′UTR of BCAR3 regulates its expressions in ovarian cancer.^47^ The knockdown of FBXO47 led to enhanced GC cell proliferation, invasion as well as migration, corresponding to overexpression of tRF‐3019a. By attenuating FBXO47, tRF‐3019a inhibitor partially inhibited GC cell proliferation, invasion and migration. ROC analysis revealed the ability of tRF‐3019a to distinguish cancer tissues from non‐tumorous tissues with AUC of 0.689. The diagnostic ability of tRF‐3019a is unsatisfactory but is better than those of CA199 (AUC = 0.585) and CEA (AUC = 0.583).^103^ The CA19‐9, CEA and CA72‐4 biomarkers are advantageous for GC screening as well as diagnosis.^104^ As a result, tRF‐3019a is potential GC diagnostic marker (Figure 6).

### 4.1 Limitations

In this review, there were three shortcomings: Firstly, all of the included studies involved patients from China. Second, there is a severe lack of high‐quality clinical studies on tRFs indicators in GC. Finally, potential publication bias and confounders which could not be avoided, because all of the studies examined had observational designs.

## AUTHOR CONTRIBUTIONS

**Maryam Kohansal:** Data curation (equal); software (equal); visualization (equal). **Ali Ghanbariasad:** Data curation (equal); software (equal); writing – original draft (equal). **Reza Tabrizi:** Conceptualization (equal); data curation (equal); resources (equal). **Abdolreza Daraei:** Investigation (equal); software (equal); validation (equal). **Mojtaba Kashfi:** Formal analysis (equal); software (equal); supervision (equal). **Hailin Tang:** Conceptualization (equal); project administration (equal); writing – review and editing (equal). **Cailu Song:** Conceptualization (equal); project administration (equal); writing – review and editing (equal). **yongming Chen:** Conceptualization (equal); project administration (equal); resources (equal).

## CONFLICT OF INTEREST

The authors confirm that there are no conflicts of interest.

## Data Availability

Data Availability Statement All authors approved that all data and materials as well as software application or custom code support our published claims and comply with field standards. Data will be made available if needed." cd_value_code="text
